# Procedural Challenges in the Analysis of Toxic Metals in Electronic Cannabinoid Delivery System Aerosols Produced for Vaping Cannabinoids

**DOI:** 10.1520/jte20250320

**Published:** 2026-09-01

**Authors:** Robert Thomas, Richard Rucker, Naudia Gray, R. Steven Pappas

**Affiliations:** 1 Scientific Solutions, 4615 Sundown Rd., Gaithersburg, MD 20882, USA; 2 Pax Labs, Inc., 660 Alabama St., 2nd Floor, San Francisco, CA 94110, USA; 3 Tobacco and Volatiles Branch, Division of Laboratory Sciences, National Center for Environmental Health, Centers for Disease Control and Prevention, 4770 Buford Highway NE, MS S110-4, Atlanta, GA 30341, USA; 4 Tobacco and Volatiles Branch, Division of Laboratory Sciences, National Center for Environmental Health, Centers for Disease Control and Prevention (retired), 4770 Buford Highway NE, MS S110-4, Atlanta, GA 30341, USA

**Keywords:** cannabis, vaping, aerosols, elemental contaminants, inductively coupled plasma-MS

## Abstract

Various methods have been used in successful and unsuccessful attempts to collect aerosols from electronic cigarettes and cannabis vaping systems over the last ten years for the purpose of assessing the possibility for potentially harmful exposures. Development of an appropriate trapping method for the analysis of metals in aerosols is one of the more challenging tasks for inorganic analytical chemists. Concentrations of many potentially harmful metal constituents of the aerosols generated by these devices are relatively low (μg/g range), so analysts must control environmental contamination of metals in trapped aerosol from the collection devices and ancillary materials used during sample preparation. The issue of contamination makes the use of materials commonly used to trap samples for organic constituents inappropriate for accurate and reliable measurement of metal constituents. In the search for appropriate trapping materials and methods, several approaches have failed because of other problems such as resistance to airflow that is necessary to activate the devices and form an aerosol. This white paper will review selected approaches to trapping aerosols for analysis of metal constituents with appropriate materials and identify problems that have been solved or that remain to be solved before the development of an ideal high-throughput method for aerosol metals analysis. Note: The incentive for this study came out of the Vape Device Safety and Testing Work Group, which is an initiative of the ASTM D37.08, Subcommittee on Personal/Household-Use Cannabis Devices and Appliances.

## Introduction

*Cannabis sativa* is federally classified as a Schedule 1 drug, although several states have legalized recreational and/or medical use. In the 2022 behavioral risk factor surveillance system, 22 states and two territories included questions for adults (≥18 years old) about cannabis use and routes of exposure.^1^ Of the respondents, 15.3 % reported current use (past 30 d).^1^ Among the current users, smoking was the most reported route of exposure (79.4 %).^1^ Vaping was reported by 30.3 % of current users, with the youngest age group (18–24 y) reporting the highest prevalence of use (44.7 %).^1^

The *Cannabis sativa* plant has over 120 known bioactive cannabinoids, predominantly produced in the flower portion of the female plant.^2^ Exposures to exogenous cannabinoids may also result in health risks that are not directly related to the cannabinoids themselves, and depend on the route and means of exposure. For example, when the exposure occurs via inhalation by smoking cannabis products, smokers are exposed to toxic products of cannabis combustion, including carbon monoxide, polycyclic aromatic hydrocarbons, and volatile and semi-volatile organic compounds (VOCs and sVOCs).^3^ The use of electronic cannabinoid delivery systems (ECDS) for inhalation of aerosol emissions may result in decreased exposures to some of the inhaled toxicants relative to exposures from cannabis combustion emissions,^4^ including toxic metals.^5^ ECDS continue to rapidly change in device design and ingredients, which could result in further unintended health consequences from vaping, such as the electronic cigarette, or vaping, product use–associated lung injury (EVALI).^6^ Therefore, it is important to monitor possible inhalation exposures to toxic substances from aerosol or smoke, including metals to ensure that the exposures pose the lowest possible health risks.

Although emitted aerosol VOCs and sVOCs are amenable to capture techniques, including solvent capture or direct analysis by solid-phase microextraction and analysis with gas chromatography–mass spectrometry (MS),^7^ aerosol sampling for toxic trace metals analysis is associated with much greater challenges since many trace metals are environmentally ubiquitous. Some of the challenges associated with the analysis of trace toxic metals in ECDS aerosols have been described in detail in a previous white paper,^8^ and therefore, not all previously discussed topics will be repeated in detail in this work. A discussion of approaches and attempts to overcome some of the challenges to the analysis of toxic metals in ECDS aerosols is provided in this white paper.

## Toxic Metals Suggested for Monitoring

Choice of toxic metals for analysis in ECDS aerosols can be guided by state regulations and evolving device component materials. Different states in the U.S. have chosen to monitor or require monitoring of various toxic metals in ECDS aerosols. The “big four” (arsenic, cadmium, lead, and mercury) have been previously discussed.^8^ Arsenic and cadmium are IARC group 1 carcinogens. Lead is an IARC group 2A carcinogen. Lead and mercury are neurotoxic. Of the “big four,” three bioaccumulate and have long biological half-lives. The toxicities and biological half-lives are justification for inclusion of these metals in monitoring products. However, an important consideration in selecting metals for monitoring is the possibility of significant exposures from a given device. Devices for delivery of nicotine and cannabinoid aerosols produced by different manufacturers are manufactured with different designs and made with different materials. The rapidly changing designs are also reflected in concentrations of metals in the aerosols. Early electronic nicotine delivery systems (ENDS) aerosol devices had internal components exposed to liquids made from brass (copper and tin), stainless steel (iron, chromium, and nickel), nichrome (chromium and nickel), and lead/tin solder, etc. Though some have reported cobalt, arsenic, cadmium, and mercury in aerosols, these metals and metalloids are less likely to be detected in aerosol since they should not be a major constituent of device materials. Cobalt and cadmium were included in the CDC analyte panel during the EVALI response and were not found in the 33 ECDS devices analyzed.^5^ It was initially assumed that metals in aerosols were derived mainly from corrosion of device components exposed to the substances in liquids or waxes used to produce aerosols.^1,9^ However, Gajdosechova et al. 2025^10^ recently identified physical damage to device components, such as metallic interconnects and/or junctions, which possibly occurred during assembly or manufacturing, as a contributor to the presence of metal and metalloid particulates in solution and in the aerosol. For that reason, publication of manuscripts describing metals in aerosols that cannot be detected in metal components of the devices should be considered suspect. As an example, one group reported cadmium in aerosols from the majority of devices they investigated. There was no apparent source of cadmium in the tested devices which could contribute to cadmium in the aerosols from these devices. However, the cadmium may have been extracted from glass rotary evaporation flasks containing acid that were used during sample preparation.^11^ Avoidance of glass in sample preparation is discussed in the following section. Table 1 shows typical metals found or analyzed in ENDS and ECDS devices with their impact on human health.

Other metals besides the “big four” have been found in ENDS aerosol device components or in substrates beneath gold alloy coated components using Scanning Electron Microscopy with Energy Dispersive Spectroscopy (SEM-EDS) and/or reported in aerosols. These metals have included chromium, nickel, copper, zinc, tin, lead, and gold,^5,9,12^ although gold was not analyzed in the aerosols. Unfortunately, existing products are subject to changes in component design and composition, and new products appear on the market with or without any approval. Analysis of all possible metals that could potentially be used in device components would be prohibitively large and challenging for a single analytical method. Some metals, such as barium, strontium, thallium, and selenium, would be a lower priority for analysis compared to other metals, such as the “big four.” Strontium’s toxicity concentration threshold is high. Barium and thallium are neurotoxic, but their thresholds for toxicity are also high and they are environmentally ubiquitous. Selenium is sometimes considered as a target for monitoring. Selenium concentrations are very low in soils, so selenium concentrations in plants from which nicotine or cannabinoids are extracted are also very low. Selenium is not considered a carcinogen except in one man-made form, selenium disulfide, which is a constituent in some topical antifungal products. However, like strontium, barium, and thallium, at elevated concentrations, selenium is toxic.

In addition to the “big four,” if additional analytes were to be added to an analytical panel, nickel might be considered a good choice. Nickel has been prevalent in components of many materials used to construct ENDS and ECDS devices over several generations. Nickel is an IARC group 1 carcinogen, neurotoxic, proinflammatory, and highly sensitizing. Copper, iron, and chromium are used to lesser degrees. Copper is also associated with a proinflammatory response. Unless the device used for vaping has very high-power settings greater than 60 W,^13^ chromium (VI) compounds are unlikely to form. Chromium may therefore be important to consider for an analyte panel, but unless the lab can show that chromium (VI) is actually formed, only total chromium will be the information obtained. Most labs do not have the instrumental capability to distinguish between chromium (III) and chromium (VI) compounds. Although arsenic is not used to manufacture ENDS or ECDS device components, the Centers for Disease Control and Prevention (CDC) tobacco products laboratory has detected arsenic concentrations in propylene glycol and glycerol at low (typically ppb) levels. Distillation of propylene glycol and glycerol from a high purity quartz flask was not sufficient to remove the low concentrations of arsenic, likely in the low-boiling arsenic acid form. Therefore, though not used in the manufacture of device components, low concentrations of arsenic may be present in liquids used in some ECDS and ENDS aerosol devices, justifying inclusion in the “big four.”

Note: It is important to understand that state-based regulations for elemental impurities in cannabis consumer products have been predominantly derived from pharmaceutical guidelines, which are mainly, an assessment of contamination from the manufacturing or delivery process. However, with the popularity of vaping devices and the multitude of different designs on the market, state regulators, in most cases, have failed to update their toxic metal limits. For that reason, state-based regulations across the US are very inconsistent. To get a better understanding of the variability of state-based metal regulations in cannabis vaping extracts/concentrates, Table 2 shows a comparison of federal limits^14,15^ for inhalation drugs with selected states for a panel of commonly regulated elemental impurities. Note: International Council for Harmonization (ICH) Quality Standard 3D (Q3D) *Guideline for Elemental Impurities* limits are regulated by the European Medicines Association.

## Solvents for Aerosol Sampling and Recovery for Analysis of Metals

Solvents including acids and diluents used for trapping and preparation of aerosol samples produced by ECDS and ENDS devices must be of the highest purity available since metal concentrations in these aerosols are low. Metals from solvents, solvent containers, and sample containers contribute to variable background contamination levels in blanks, calibration standards, quality control (QC) samples, and product samples. Ultrapure water with greater than 18 MΩ·cm resistivity should always be used for metals analysis to minimize contamination of samples and standards with metals. Acids should be ultrapure grade obtained in high-purity fluoropolymer bottles or distilled in an appropriate fluoropolymer or high-purity quartz distillation system. “Trace metals grade” acids obtained in glass bottles should not be used since hydrogen ion exchanges with metal contaminants that are mobile in glass matrices. “Trace metals grade” acids may be high purity before bottling but will not maintain low metal contaminant levels for long after being bottled in glass. Even organic solvents have metal contaminants that may interfere with accuracy and precision of analyses at trace metals concentrations such as exist in ENDS and ECDS aerosols. When organic solvents are used, electronics grade VLSI solvents obtained in high purity HDPE/LDPE, polypropylene, or fluoropolymer bottles should be the minimum purity acceptable, although ULSI grade is preferable if available. When a necessary solvent is unavailable in these purities, it may be purified by distillation from high-purity quartz flasks,^5^ but even distillation may not sufficiently purify some solvents free of arsenic in the form of low-boiling point arsenic acid. Table 3 shows some common materials used for lab-based volumetric ware and their respective typical elemental composition.^16^

## Approaches to Aerosol Sampling and Recovery for Analysis of Metals

The first consideration in sampling aerosols for the analysis of metals is the form in which the metals are present in the aerosol. Metals in aerosols from ENDS and ECDS are in a dissolved ionic form, to some degree, but are predominantly in poorly soluble particles derived either from oxidative corrosion, machining/cutting of internal device components, or physical damage to metallic connections and/or junctions that are exposed to the liquid and waxy or solid substances that are vaporized.^10,11,17^

A second consideration in the analysis of metals in aerosols is the composition of the liquid in the aerosol droplets. ENDS aerosols are predominantly hydrophilic since nearly all ENDS liquid solvents/fillers consist of propylene glycol, glycerol, water, or a mix of the three.^18^ After trapping, these aerosols and particulate metal compounds may be dissolved during sample recovery and dilution with an acid mixture suitable for use with inductively coupled plasma-MS (ICP-MS).^9^ ICP-optical emission spectroscopy may be suitable for analyses of some metals present at relatively high concentrations. ECDS aerosols are similar in composition to their parent formulations, which are composed predominantly of hydrophobic liquids or waxy substances including neat cannabinoid extracts or extracts formulated with various terpenes and plant oils; although in the past other solvents such as medium-chain triglycerides, vitamin E acetate, or other tocopherols (found in illicit markets), or emulsions in hydrophilic propylene glycol and glycerol have also been manufactured and sold.^19–21^ The variability in the types of aerosols emitted by various ECDS devices complicates the analysis of metals in the aerosols. The analyst must take into account whether the aerosol trapping method quantitatively recovers the metal-containing particles in the aerosol that might be embedded in hydrophobic waxy substances or liquid droplets and whether they will be lost in the introduction system or be liberated in the solution that enters the plasma. The chemical nature of the aerosolized substances must either be known in advance or the analytical method for metals must be designed to take into account the likelihood of hydrophobic constituents of the aerosol as well as the possibility of hydrophilic constituents such as propylene glycol and glycerol, and, of course the metal compounds. Most metals and metal oxides in the aerosols are poorly soluble or insoluble in ultrapure water and in organic solvents in the absence of an appropriate acid mix or chelating agents.

**FIG. 1:**
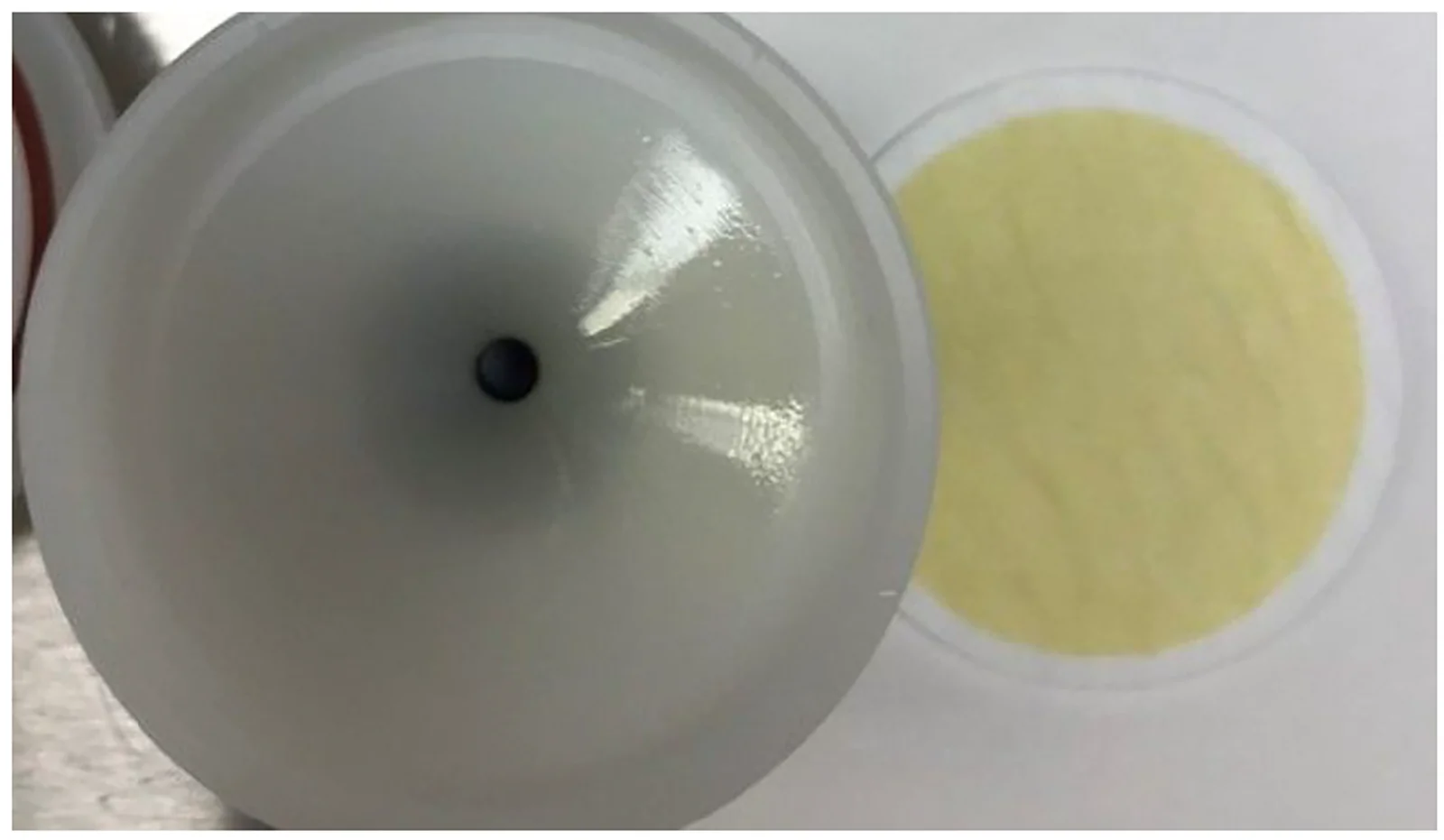
Low-purity quartz/glass fiber filters should be avoided because of high extractable metals and fines background. This image was created by the authors from the Centers for Disease Control and Prevention and is not publicly available.

A third consideration in the analysis of metals in ECDS aerosols is the design of the device used to trap the variety of aerosol compositions, with consideration of good inorganic analytical practices, aerosol recovery, and its compatibility with the solvents used to recover aerosols from the trap. Glass fiber filters, impingers, round bottom and rotary vaporization flasks, beakers, tubing, watch glasses, and autopipettor syringes, etc., should be completely avoided when performing trace metal sample preparation, especially in the presence of acids. The same is true for low-purity quartz filters and syringe barrels, etc.^22^ Sample preparation materials and techniques that should be avoided and good inorganic analytical practices for aerosol metals analyses have been reviewed.^23,24^ It should be noted that good inorganic analytical practices may not be a term that ICP-MS practitioners are familiar with unless they work routinely in the ultra-trace environment. It refers to an understanding and specific application of good practices as it pertains to ultra-trace analysis of elemental analytes; namely, selection and use of well-characterized materials with consistent and controlled elemental profiles, which are fit for this demanding type of analysis. A common commercially available quartz filter is shown in figure 1.

**FIG. 2:**
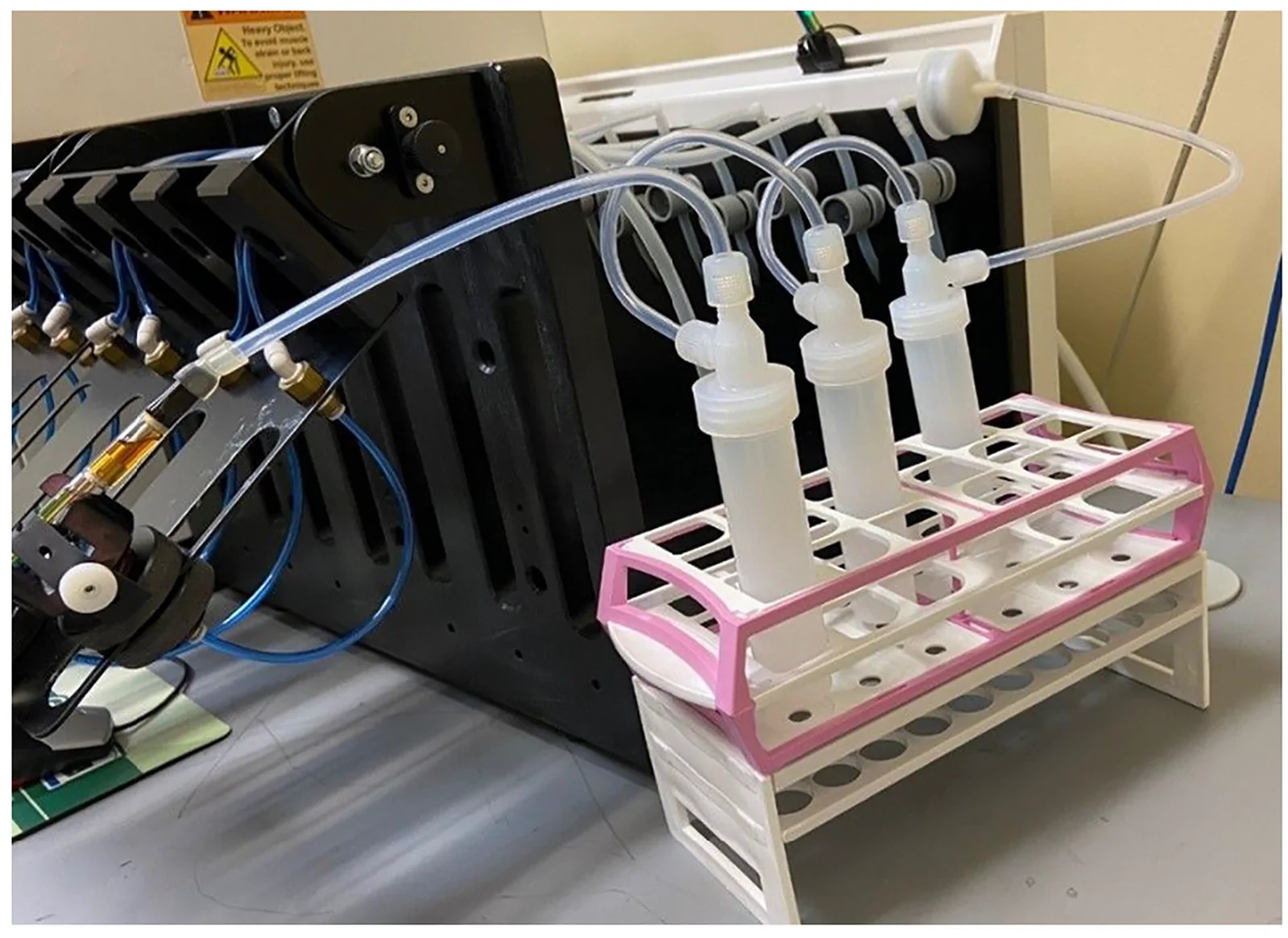
A series of three 60 mL PFA impingers with 4 mm × 1 mm holes near the bottom of each aerosol inlet tube beneath the liquid levels permit capture of aerosols in 3 mL × 30 mL liquid. If impingers are more than half filled, liquid is lost because of carry-over beyond the impinger system. This image was created by the authors from the Centers for Disease Control and Prevention and is not publicly available.

Very little has been published on trapping of ECDS aerosols for metals analyses. One group^25,26^ reported quantitatively trapping hydrophobic ECDS aerosols using two fritted glass impingers containing 25 mL of liquid at a 25 mL/s airflow rate, which may be more consistent with the vaping topography of those who vape cannabinoids than the 18.3 mL/s flow rate prescribed in International Organization for Standardization (ISO) standard 20768. In the first of their impinger only strategies, two impingers contained 8 % nitric acid and 2 % hydrochloric acid in ultrapure water,^25^ however the authors stated that oil droplets from ECDS aerosols clogged the frits when only aqueous solvent was used. In the second strategy, the first impinger contained acetone^26^ to prevent the oil droplets from clogging the impinger frit. The use of impingers with sufficient liquid volume to trap metals is an acceptable practice. However, although it is possible that the use of frits enabled quantitative aerosol trapping in two impingers containing 25 mL of liquid, the use of glass containers and impingers with acids is only appropriate for mercury, which is typically at very low concentrations in glass, and for other metals at high concentrations since metals in glass silicate matrices contaminate the sample solution. Data obtained with poor inorganic practices such as using glass flasks, digestion tubes, and impingers with acids should be considered suspect and unreliable. For that reason, in our experience, the use of glass and quartz impingers/filters should be avoided. Even though they are commercially available, they are not of sufficiently high purity to be used in this type of demanding analysis. One approach to avoid this problem is to use an impinger made from perfluoroalkoxy (PFA) and to modify it by drilling holes to produce smaller bubbles, which will help in solvation by the capture solvent (described hereafter). A trapping design using multiple low-metal PFA impingers is shown in figure 2.

High purity PFA impingers are available from at least one manufacturer. The Tobacco Products Laboratory at CDC determined that a minimum of 3 mL × 30 mL phosphate-buffered saline or dimethyl sulfoxide (DMSO) in 60 mL PFA open-ended impingers with 4 mm × 1 mm holes drilled in the bottoms of air transfer tubes were required to quantitatively trap total mainstream smoke particulate matter (TPM) from combustible tobacco products provided to the U.S. Food and Drug Administration Toxicology Laboratory for total smoke cell culture studies. The TPM samples were obtained using the International Organization for Standardization (ISO) Standard 3308 and World Health Organization Method Number 1 smoking regimes, which average 17.5 and 27.5 mL/s average airflow rates, respectively, in a bell-shaped puff profile. Recovery of TPM was quantitative in DMSO, but the hydrophobic components of TPM were difficult to recover quantitatively from impinger walls with PBS.

**FIG. 3:**
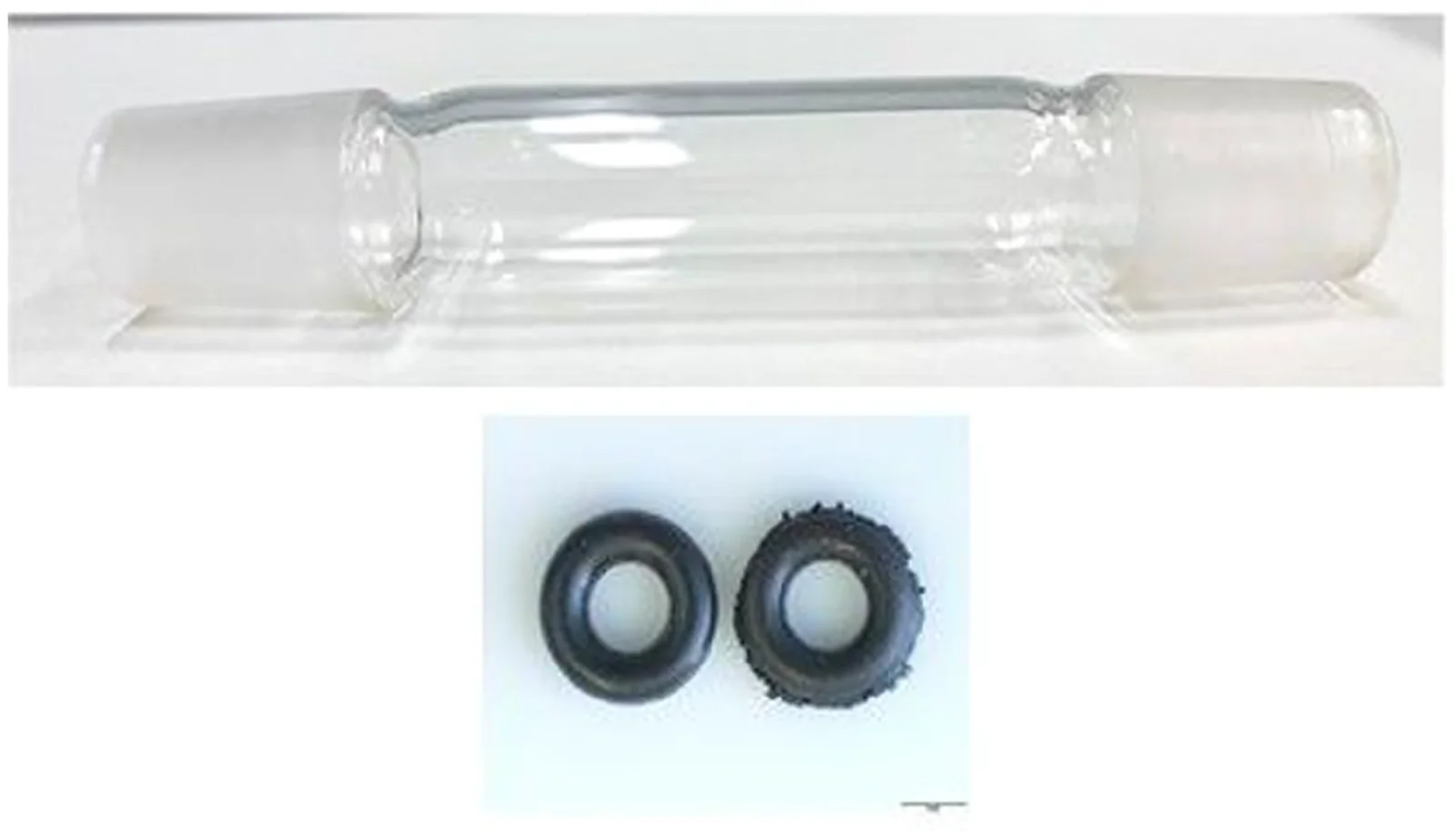
Electrostatic precipitator showing degradation of neoprene seals by propylene glycol leading to inconsistent and low aerosol recoveries. This image was created by the authors from the Centers for Disease Control and Prevention and is not publicly available.

The Tobacco Products Laboratory at the CDC attempted to recover ENDS aerosols for analysis of metals using a Borgwaldt (Körber Technologies, Hamburg, Germany) LX5.1 vaping machine to produce ENDS aerosols for trapping inside 29 joint, 20 mm internal diameter ultrahigh-purity quartz precipitation tubes, using electrostatic precipitation with a 24 kV tungsten probe potential. Unfortunately, recovery of the aerosol, which was calculated by dividing the mass gained in the EP tube by the mass lost in the vaping device, was poor and variable, ranging from 20 % to 84 % depending on the composition of the ENDS liquid in a given device. This was likely attributed to the fact that at the rate of airflow through the quartz tube, there was insufficient time for all of the vapor to condense to form liquid droplets, especially for the lower-boiling solvents. An additional problem was that the ends of the electrostatic precipitation tubes were sealed with neoprene seals that were attacked by propylene glycol, which is commonly used in both ENDS and ECDS liquids. The propylene glycol weakened seals ruptured during aerosol collection inside the tubes at high potential necessary for trapping the aerosols and contaminated the collected aerosol with numerous neoprene particles. An electrostatic precipitator used in this study is exemplified in figure 3, showing degradation of the neoprene seals by propylene glycol.

Further work with this approach was discontinued. Authors associated with Cerulean^27^ (Milton Keynes, UK) described in a white paper the use of a Cerulean CETI1 vaping machine with an electrostatic precipitation unit set at 20 kV inside a high-purity quartz tube for trapping aerosol from an ECDS device. They also reported an unsatisfactory, approximately 40 % aerosol mass recovery and stated that the efficiency of recovery of this type of aerosol was less than would be expected for cigarette smoke and within the range of recoveries of ENDS aerosols, in agreement with CDC observations.

Gajdosechova and Marleau-Gillette^17^ analyzed metal corrosion particles in ECDS device liquids with oxygen addition to the plasma after dilution in Premisolv. However, this approach had not been successfully applied to quantitative analysis of metals in aerosols until very recently, when Gajdosechova et al. published a study in which metal particles were detected in the aerosol.^11^ In the Tobacco Products Laboratory at CDC, an approach using organic-mode ICP-MS (2024)^17^ was unsuccessfully adapted from particle analysis to the quantitative analysis of metals in ECDS aerosols. Unfortunately, the metal stabilizing solution that contains hydrophobic chelating agents required for preparation of dissolved metals in mineral oil standards with a petroleum-based ICP solvent like that of Premisolv, had excessive metal contamination. The stabilizer elevated the background of all metals and caused poor linearity, particularly with cobalt, nickel, and cadmium. However, preparation of commercially available metal in mineral oil standards diluted in a petroleum based ICP solvent without the stabilizer did not provide the required calibration linearity, particularly with low concentration cobalt calibration standards (Note: Our lowest standard for the 10 metals ranged from 0.04 to 8 ppb and the highest calibration standards ranged from 1 to 200 ppb depending on the metal). Without the stabilizer, the standards were not stable in solution, as was observed by rapidly deteriorating linearity on subsequent days. As a result, we did not further pursue this approach for analyses at the trace metal concentrations found in various hydrophobic ECDS aerosols. We concluded that if the stabilizer had to be added to maintain metals in suspension, the contamination that came with the stabilizer—although acceptable for single-particle ICP-MS, where the metal particles are detected as transient peaks above the baseline—was not acceptable for determining trace metals in ECDS aerosols quantitatively using conventional sample introduction to detect analyte concentrations with steady signals.

**FIG. 4:**
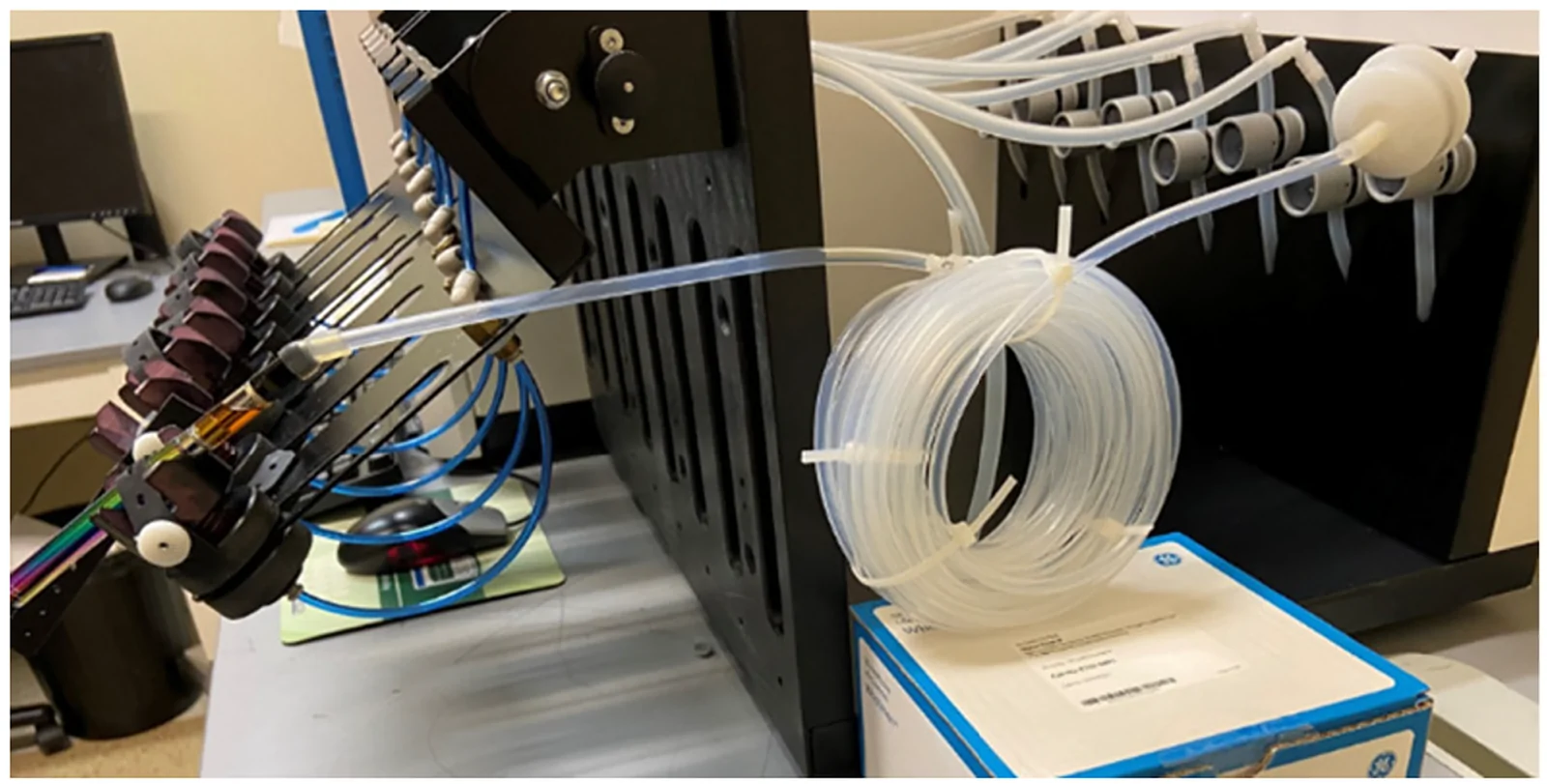
FEP tubing trap setup for the DEGMEE solvent method described, using a Cerulean CETI-8 smoking machine. This image was created by the authors from the Centers for Disease Control and Prevention and is not publicly available.

Gonzalez-Jimenez et al.^5^ used long (518 cm and 3.97 mm internal diameter (i.d.)) high-purity fluorinated ethylenepropylene (FEP) tubing to trap metals-containing aerosols from ECDS devices, during the EVALI response during which limited volume of sample in the device allowed for only 15 puffs to be collected. Further research using our preferred 50 puff collection showed that increasing the tubing length to 914 cm provided better aerosol recovery for a broader variety of aerosol matrices produced by ECDS devices since that time. Condensed aerosols with metals were first rinsed from the tubing with high-purity quartz-distilled diethylene glycol monoethyl ether (DEGMEE), a good organic solvent that is also miscible with water, for simple dilution to avoid increasing sample preparation time with microwave digestion. The DEGMEE rinse was followed with four 2 % v/v nitric acid + 1 % v/v hydrochloric acid rinses to assure that any metal oxide particles left behind by the organic solvent were dissolved and rinsed from the tubing. Although this trapping method is consistent with good inorganic analytical practices, one weakness of the technique is the skill required to prevent sample contamination with environmentally ubiquitous metals. The organic solvent also must be quartz distilled and stored in a high-metal-purity polymer bottle without contaminating it, adding complexity to preparation. It would be easy for an inexperienced analyst to lose sample or contaminate solvents or samples during rinses from the tubing. Finally, the time required for acid, ultrapure water, and high-purity organic solvent precleaning and drying of the tubing, as well as the time required to carefully rinse the sample from the long length of tubing, renders the technique low in sample throughput, and therefore not ideal for high-throughput regulatory compliance and QC laboratories. The FEP tubing trap set up for this approach using a Cerulean CETI-8 smoking machine is shown in figure 4.

## Attempts to Improve Analytical Throughput in ECDS Aerosol Sample Preparation

In the Tobacco Products Laboratory at CDC, several alternatives to fluoropolymer tubing have been tested in attempts to improve sample preparation throughput.

Polytetrafluoroethylene (PTFE) membrane filters were tested. When 47 mm PTFE membrane filters with 1.0 μm pore size were tested, it was determined that the filters met the ISO standard 20768 rectangular puff profile specification during the first 3 to 4 puffs. However, after the 4^th^ puff, the filters became clogged with liquid or waxy extracts and failed to meet the puff profile specifications because of increased airflow resistance through the filters. No further testing on aerosol collection or metals background was pursued with these filters.

PTFE depth filters were also tested. When 47 mm PTFE depth filters with a 4 μm pore size were tested, they met the ISO standard 20768 rectangular puff profile specification throughout 50 puffs. Unfortunately, these depth filters were found to have metal contamination that varied from filter to filter. Attempts to preclean the depth filters by repeatedly soaking overnight in dilute acid (typically 2–5 % nitric acid), sonicating in acid with and without heat, failed to eliminate the metal contaminants from the filters. A microwave digestion precleaning step (typically 170–200°C for 15–20 min) was required to reduce extractable metals concentrations for some of the filters below our aerosol trace metal calibration standards. This likely indicated contamination with metal particles in pores that were poorly available to acid rinses, but which were somewhat better dissolved during microwave filter digestions. However, the microwave filter digestions caused deformation of the filters, which made them difficult to fit correctly into the filter holders. The pretreated filters were digested again along with samples for use as digestion blanks. The twice-digested filter blanks still sporadically had metals backgrounds that were higher than our lowest standards. The elevated extractable metals available even after multiple digestions or overnight soaks suggested that depth filters were unreliable and that additional microwave digestions to prepare filters for use with aerosol samples was impractical.

One manufacturer made PTFE fiber wool filters in polypropylene cartridges available. The Tobacco Products Laboratory at CDC tested these filters intact as well as the PTFE fiber wool separately. The intact filters were found to contain variable metal contamination, which diminished, then reappeared after repeated acid rinses and overnight soaks. The manufacturer concluded that the contamination was likely because of metal wear shavings from machinery used to cut the PTFE fiber wool and obtained the material from a different manufacturer. Contamination was found to be lower in a few subsequent samples tested but was still inconsistent from sample to sample. One possibility to explain the variability was that like the depth filters, air pockets that were not reached by acid solvent during repeated pre-rinses had metal particles that were reached in subsequent rinses. It is hoped that the problem with contamination from production machinery will be solved, but for the present, pursuit of this approach was also discontinued.

## Comparative Assessment of Different Methodologies

Table 4 represents a summarized list of the pros and cons of all the methodologies discussed in this white paper, with an assessment of their detection capability, ease of use, and sample throughput. This is not meant to be a comparison of the performance characteristics offered by the different analytical procedures described but is intended to be a practical guideline for researchers who are interested in developing a robust procedure to better understand of what metals might be leaching into the vape liquid and the resulting vaping aerosol. With so many different metal components used in the multitude of devices manufactured today, it would be extremely difficult to come up with a list of performance characteristics for all the different configurations that have been evaluated and published in the open literature.

## Conclusions and Future Perspectives

Because of the low concentrations of metals in aerosols obtained from cannabis vaping devices, all solvents and materials used for trapping aerosols for analysis must be repeatably of high purity with respect to leachable metals. This precludes the use of glass or low-purity quartz materials, especially when an acid solution is in contact with the materials. The trapping device must permit sufficient airflow to achieve specified rectangular puff profiles at the airflow rate specified for the number of puffs required to produce a sufficient amount of aerosol for analysis. The search to find such materials has presented an ongoing challenge.

At least one method that meets requirements for good inorganic analytical practices has been published. However, recovery of aerosol samples from the high purity condensation tubing used to trap samples requires skill to recover the samples without contamination and is inconsistent with the high-throughput requirements of regulatory and QC laboratories. Continued effort to solve the throughput problem or to ensure availability of consistently pure trapping materials is required.

We are hopeful that the work outlined in this white paper will lead to an ASTM D37 Standard or Guideline which will address many of the challenges outlined in this study and eventually become a robust analytical method capable of being utilized by cannabis testing labs to better understand the mechanism of aerosol generation, resulting in safer cannabis vaping consumer products. The start of this standardization process will begin once this white paper is published.

It is also important to mention that recent events to reschedule cannabis from Schedule 1 to Schedule 3 have been initiated by executive order by the President. Although it is uncertain what this means long term, it should enable more research into cannabis vaporization as an administration method. In particular, this would hopefully include emissions studies performed at state and federal levels, which will seek to quantify the types and amounts of byproducts and contaminants to which users may be exposed to when using ECDS. This review/white paper will aid researchers’ evaluations of the current limited methodology available for this type of analysis.

## Figures and Tables

**TABLE 1:** Typical metals analyzed in ENDS and ECDS devices and their impact on human health

Metal in Device Component	Toxicity/Impact on Health
Aluminum: kanthal	Pulmonary irritant and neurotoxic
Chromium: nichrome and stainless steel	Chromium (III): sensitization
Iron: stainless steel and hastelloy	Pulmonary irritant and redox
Cobalt: metal impurity	Group 2a and 2b carcinogen
Nickel: nichrome, stainless steel, hastelloy, and other alloys	Group 1 carcinogen
Copper: brass and connectors	Irritant and redox active
Arsenic: potential contaminant in Propylene Glycol/Vegetable	Group 1 carcinogen
Glycerin (PG/VG) solvents used in ENDS devices; “big four element”	
Cadmium: “big four element”	Group 1 carcinogen
Tin: solder and exposed substrate metal impurity	Particle inflammation
Lead: solder and exposed substrate metal impurity; “big four element”	Group 2a carcinogen

**TABLE 2:** Comparison of federal limits for inhalation drugs with selected states for a panel of elemental impurities

	Federal Limits for Inhalation Drugs, μg/g	Vast Majority of US States, μg/g	Maryland, μg/g	Michigan, μg/g	New York, μg/g
Lead	0.5	0.5	1.0	0.5	0.5
Cadmium	0.3	0.3	0.4	0.2	0.2
Arsenic	0.2	0.2	0.4	0.2 (Inorganic)	1.5
Mercury	0.1	0.1	0.2	0.1	0.3
Chromium	0.3	N/R	0.6	0.6	110
Copper	3.0	N/R	N/R	3.0	30
Nickel	0.6	N/R	N/R	0.5	2.0
Antimony	2.0	N/R	N/R	N/R	2.0

*Note*: Units are expressed in (μg/g), based on a 10 g sample, except for Maryland which is 5 g and New York which is 1 g. N/R = not required.

**TABLE 3:** Common laboratory volumetric ware materials with major elemental impurities

Material	Number of Elements	Total ppm	Major Impurities
Polystyrene (PS)	8	4	Na, Ti, and Al
Tetrafluoroethylene (TFE)	24	19	Ca, Pb, Fe, and Cu
Low density PE (LDPE)	18	23	Ca, Cl, K, Ti, and Zn
Polycarbonate (PC)	10	85	Cl, Br, and Al
Polymethyl pentene (PMP)	14	178	Ca, Mg, and Zn
Fluorinated ethylene propylene (FEP)	25	241	K, Ca, and Mg
Borosilicate glass	14	497	Si, B, and Na
Polypropylene (PP)	21	519	Cl, Mg, and Ca
High density PE (HDPE)	22	654	Ca, Zn, and Si

*Note*: Reprinted with permission from Moody and Lindstrom^16^, American Chemical Society.

**TABLE 4:** Performance summary of the capabilities of the methodologies discussed in this white paper

Category	Techniques	Performance Characteristics 1–5 (1—worst and 5—best)	Advantages	Disadvantages	Further Reading
Filtration	Membrane filter (PTFE, and MCE, etc)	Detection capability—2 Ease of use—3 Throughput—3	Ease of setup and low background levels possible	Low sample loading (~5 mg) before increased pressure drop alters puff profile rapidly	Palazzolo et al. (2017)^28^
	Quartz fiber filter	Detection capability—1 Ease of use—3 Throughput—3	Ease of setup and high sample loading (~100 mg)	Filter to filter variability and high metal background levels	Saffari et al. (2014)^29^
	Depth filter	Detection capability—2 Ease of use—2 Throughput – 2	Very high sample loading	Irreproducible results obtained owing to difficulty to fully clean	Colligan (2023)^30^
Electrostatic capture		Detection capability—2 Ease of use—1 Throughput—2	Commercially available equipment	Low reproducibility and recovery observed	Tindall and Taylor (2022)^27^
Solvent/liquid capture	Impinger	Detection capability—3 Ease of use—2 Throughput—3	High sample loading	Large bubble size and high breakthrough if aqueous solvent is used	Mallampati, McDaniel, and Wise (2021)^25^ Gajdosechova et al. (2025)^11,17^
	Fritted bubbler	Detection capability—1 Ease of use—2 Throughput—3	High sample loading and small bubble size	Multiple bubblers required to prevent clogging—difficult to clean	McDaniel, Mallampati and Wise (2021)^26^
Condensation	FEP tubing	Detection capability—4 Ease of use—1 Throughput—2	Low metal blanks. Potentially offers the lowest limits of quantitation	High operator skill required to prevent sample contamination Not ideally suited for high sample throughput	Gonzalez-Jimenez et al. (2021)^5^

*Note*: Ratings are based on the authors’ qualitative assessment and observations rather than on side-by-side quantitative validation.

## Data Availability

No data, models, or code were generated or used for this study.
